# Dietary Vitamin C, E and β-Carotene Intake Does Not Significantly Affect Plasma or Salivary Antioxidant Indices and Salivary C-Reactive Protein in Older Subjects

**DOI:** 10.3390/nu9070729

**Published:** 2017-07-09

**Authors:** Anna Gawron-Skarbek, Agnieszka Guligowska, Anna Prymont-Przymińska, Małgorzata Godala, Agnieszka Kolmaga, Dariusz Nowak, Franciszek Szatko, Tomasz Kostka

**Affiliations:** 1 Department of Hygiene and Health Promotion, Medical University of Lodz, Hallera St. 1, Łódź 90-647, Poland; franciszek.szatko@umed.lodz.pl; 2 Department of Geriatrics, Medical University of Lodz, Pieniny St. 30, Łódź 90-993, Poland; agnieszka.guligowska@umed.lodz.pl (A.G.); tomasz.kostka@umed.lodz.pl (T.K.); 3 Department of General Physiology, Medical University of Lodz, Mazowiecka St. 6/8, Łódź 92-215, Poland; anna.przyminska@umed.lodz.pl; 4 Department of Hygiene of Nutrition and Epidemiology, Medical University of Lodz, Hallera St. 1, Łódź 90-647, Poland; malgorzata.godala@umed.lodz.pl (M.G.); agnieszka.kolmaga@umed.lodz.pl (A.K.); 5 Department of Clinical Physiology, Medical University of Lodz, Mazowiecka St. 6/8, Łódź 92-215, Poland; dariusz.nowak@umed.lodz.pl

**Keywords:** plasma total antioxidant capacity, saliva, uric acid, C-reactive protein, diet, vitamin C intake, vitamin E intake, β-carotene, DPPH, FRAP

## Abstract

It is not clear whether habitual dietary intake influences the antioxidant or inflammatory status. The aim of the present study was to assess the impact of antioxidative vitamins C, E, and β-carotene obtained from daily food rations on plasma and salivary Total Antioxidant Capacity (TAC), uric acid and salivary C-reactive protein (CRP). The study involved 80 older subjects (66.9 ± 4.3 years), divided into two groups: group 1 (*n* = 43) with lower and group 2 (*n* = 37) with higher combined vitamins C, E and β-carotene intake. A 24-h dietary recall was obtained from each individual. TAC was assessed simultaneously with two methods in plasma (Ferric Reducing Ability of Plasma—FRAP, 2.2-diphenyl-1-picryl-hydrazyl—DPPH) and in saliva (FRAS and DPPHS test). Lower vitamin C intake corresponded to higher FRAS. There were no other correlations between vitamins C, E or β-carotene intake and antioxidant indices. Salivary CRP was not related to any antioxidant indices. FRAS was decreased in group 2 (*p* < 0.01) but no other group differences for salivary or for plasma antioxidant parameters and salivary CRP were found. Habitual, not extra supplemented dietary intake does not significantly affect plasma or salivary TAC and salivary CRP.

## 1. Introduction

There are numerous interventional studies assessing the potential influence of various nutritional compounds added to food [1,2] or beverages [3] in Daily Food Rations (DFR) on antioxidant capacity. Increased antioxidant status has been associated with high consumption of fruit, vegetables and plant oils as main food sources of antioxidative compounds [4,5]. Vitamin C, E and β-carotene are representative dietary antioxidants so their high content in the DFR is expected to enhance antioxidant potential in body fluids, cells and tissues. However, limited information is available on whether different antioxidant capacities found in different body fluids reflect a habitual dietary intake of antioxidants [6,7]. Subjects following a naturally antioxidant-rich diet might experience different biological effects than individuals being supplemented by multivitamins and minerals [8]. There are many external (diet, cigarette smoking) and internal (biochemical disorders) factors that might affect the final result, and preclude an unequivocal conclusion of whether habitual dietary intake without any special regimens is also associated with higher antioxidant status. Oxidative stress and inflammatory conditions are inter-related [9,10]. One of them may appear before or after the other, but the two usually occur together, resulting in both of them taking part in the pathogenesis of many chronic diseases. Complex biochemical interactions between pro- and antioxidants result in a relatively stable homeostasis state. It may be generally assumed that the inflammatory indices and accompanied prooxidants are low when the systemic antioxidant potential is strong enough to counteract these undesirable conditions. Dietary modification may affect inflammatory processes and protect against chronic diseases [11]. It is thought that the protective effects of fruit and vegetable consumption result from the presence of low-molecular antioxidants such as α-tocopherol, ascorbic acid, or β-carotene, as well as non-vitamin antioxidants, such as polyphenols and anthocyanins, or from the synergy of several different antioxidant compounds [5]. Other reports indicate that vitamin C, especially in doses exceeding daily recommended dietary allowance, may exert a prooxidant effect [12].

C-reactive protein (CRP) is an acute-phase protein that increases during inflammatory disorders [13,14]. CRP has been identified as a hallmark of systemic inflammation and is used as a risk bio-marker of different health conditions: cardiovascular disease [15], periodontitis [16,17], metabolic syndrome or diabetes mellitus [18]. Usually it is assessed in plasma but new research attitude appeals to noninvasive CRP or antioxidant parameters determination techniques using saliva samples [19]. Saliva may represent an alternative means for evaluation of the impact of dietary antioxidant intake on the plasma antioxidant defense system.

The variety of methods assessing antioxidant defense system provides a range of results which are at times inconsistent. The assessment of Total Antioxidant Capacity (TAC) may be a better approach than determining the capacities of individual antioxidants. An increased antioxidant capacity in body fluid may not necessarily be a desirable condition if it reflects a response to increased oxidative stress/inflammation. Similarly, a decrease may not necessarily be an undesirable condition if the measurement reflects decreased production of reactive species. These complications suggest that a “battery” of measurements is going to be more sufficient to adequately assess oxidative stress, as well as the antioxidant barrier level, in biological systems than any single measurement of antioxidant status [20]. The content of Uric Acid (UA), the strongest endogenous antioxidant, contributing about 70% of plasma and salivary TAC [21,22], should also be taken into consideration.

The aim of the study was to assess the impact of nutrients, mostly the antioxidative vitamins C, E and β-carotene, obtained from DFR on plasma and salivary TAC, UA and salivary CRP in older adults.

## 2. Materials and Methods

### 2.1. Patients

The study was carried out in 80 patients (66.9 ± 4.3 years), 86% of whom were females. The subjects had been treated in Outpatient Geriatric Clinic of the Medical University of Lodz (Łódź, Poland) and selected from a group of subjects participating in the healthy lifestyle workshops organized under the governmental program for the Social Activity of the Elderly (2014–2020) who volunteered to undergo a detailed dietary and laboratory (blood plasma and saliva) assessment. The subjects were consecutively recruited based on inclusion criteria and combined value of vitamin C, E and β-carotene intake (see below) in order to obtain balanced sex composition of the two groups, differing in combined intake value of antioxidant vitamins.

Some patients suffered from hypercholesterolemia (*n* = 48), arterial hypertension (*n* = 39), osteoarthritis (*n* = 33), thyroid insufficiency (*n* = 26), osteoporosis (*n* = 19), duodenal and gastric conditions (*n* = 14), diabetes mellitus (*n* = 14) and heart failure (*n* = 11). All diagnosed diseases were in stable phase and pharmacologically controlled. The treatment usually involved angiotensin-converting enzyme inhibitors (*n* = 25), levothyroxine (*n* = 26), statins (*n* = 23), diuretics (*n* = 22), beta-blockers (*n* = 18), aspirin (*n* = 17), calcium channel blockers (*n* = 9), proton pump inhibitors (*n* = 7), oral antidiabetic drugs—metformin (*n* = 9) and sulfonylureas (*n* = 6).

None of the subjects was diagnosed with tobacco addiction, active inflammatory processes (plasma CRP < 3 mg∙L^−1^), renal dysfunction, disability or dementia. None used any special diet. The study had been approved by the local ethics committee (RNN/73/15/KE) and informed consent was obtained from each subject. The investigations were carried out following the rules of the Declaration of Helsinki of 1975, revised in 2008.

### 2.2. Study Protocol and Measurements

The examinations took place in the Department of Geriatrics and the laboratory measurements were performed in the Department of Clinical Physiology, in the Central Scientific Laboratory and in the University Hospital and Educational Center, all at the Medical University of Lodz. The subjects reported to the Center between 8.00–10.00 a.m. after overnight fasting and rest for at least 12 h before blood and saliva collection. The time window between teeth cleaning and non-stimulated saliva sample collection was never shorter than 1.5 h. A comprehensive assessment, including age, sex, drug use, smoking and dietary habits was performed with each subject [23]. A 24-h dietary recall from the day before the examination was obtained from each individual.

#### 2.2.1. Anthropometric Data

Height and weight were measured and the Body Mass Index (BMI) was calculated (overweight was for BMI in the range 25–30 kg∙m^−2^, obesity for BMI over 30 kg∙m^−2^). Measurements of waist and hip circumference were taken and Waist-to-Hip Ratio (WHR) was computed as an index of visceral obesity (diagnosed if WHR > 0.8 in females or >1.0 in males).

#### 2.2.2. Plasma UA, CRP and Lipid Profile Determinations

Blood samples (about 9 mL) were drawn from the antecubital vein and collected for further TAC measurements into Vacuette tubes with lithium heparin or into vacutainer tubes with K3 EDTA for other tests (Vacutest, Kima, Italy). Thereafter the samples were incubated for 30 min at 37 °C and then centrifuged (10 min, 4 °C, 2880× *g*). The resultant plasma samples for TAC measurements (approximately 4 mL) were stored at −80 °C, for no longer than three months [24,25] and the rest was used to assess UA, CRP concentration and lipid profile parameters.

Enzymatic methods were used to determine plasma total cholesterol (TCh), triglycerides (TG) and UA concentration (BioMaxima S.A. diagnostic kit, Lublin, Poland with Dirui CS-400 equipment). High-density lipoprotein cholesterol (HDL-Ch) was measured by the precipitation method (BioMaxima S.A. diagnostic kit). Low-density lipoprotein cholesterol (LDL-Ch) was estimated using the Friedewald formula. Plasma CRP was measured by immunoassay (BioMaxima S.A. diagnostic kit, Lublin, Poland with Dirui CS-400 analyzer, Jilin, China).

#### 2.2.3. Plasma TAC

Plasma TAC measurements were performed using two spectrophotometric methods: Ferric Reducing Ability of Plasma (FRAP) [21] with some modifications [24], and 2.2-diphenyl-1-picryl-hydrazyl test (DPPH) [24,25]. The details of both methods are described elsewhere [24,26].

#### 2.2.4. Salivary TAC

Saliva samples (approximately 5mL) were centrifuged to separate all debris (10 min, 4 °C, 1125× *g*) [27]. The supernatant was stored at −80 °C max. for 30 days. Salivary TAC also was measured spectrophotometrically using the same equipment (Ultrospec III with Spectro-Kinetics software—LKB Biochrom Pharmacia, Cambridge, UK) and two methods, as for plasma TAC. For Ferric Reducing Ability of Saliva (FRAS) 120 μL of saliva were added to 900 μL of FRAS reagent, but deionized water was not used.

For the 2.2-diphenyl-1-picryl-hydrazyl test of saliva (DPPHS), as for DPPH [24], 200 μL of saliva was required for the deproteinization process; however, for the singular assay, 25 μL of deproteinized saliva were added to 975 μL of DPPH reagent mixture.

To enhance the data reliability, all results were calculated as a mean from three separate experiments. The salivary and plasma TAC assays were performed within the same time frame.

#### 2.2.5. Salivary UA

Salivary UA (SUA) was analyzed using the MaxDiscovery™ Uric Acid Assay Kit (Bioo Scientific, Austin, TX, USA). Hydrogen peroxide, liberated by the action of uricase, reacted with a chromogenic dye using peroxidase to form a visibly colored (red) dye product. The absorbance was measured at 520 nm and the result was proportional to SUA concentration [28].

#### 2.2.6. Salivary CRP

The salivary CRP assays (ELISA Kit—Salimetrics, PA, USA) were based on the colorimetric CRP peroxidase reaction on the substrate tetramethylbenzidine. Optical density was read on a standard VICTOR^TM^ ×4 multifunctional microplate reader (Perkin Elmer, Waltham, MA, USA) at λ = 450 nm. The amount of CRP peroxidase detected was directly proportional to the amount of CRP present in the saliva sample [29].

#### 2.2.7. Nutritional Evaluation

A 24-h recall questionnaire was used to register and then encode the intake of food, beverages, and supplements during the preceding day. The intake of energy, nutrients, vitamins, minerals in the DFR was calculated using the Diet 5.0 software package (developed by the National Food and Nutrition Institute, Warsaw, Poland) and compared with recommendations [30,31]. The degree of insufficient intake of analyzed antioxidative vitamins was estimated according to the following age and sex standards: EAR (the Estimated Average Requirement) for vitamin C (<60 mg/<75 mg, for females/males respectively) and AI (the Adequate Intake) for vitamin E (<8 mg/<10 mg) [30]. No dietary advice was given for the cases before a 24-h recall.

A further extra comparative analysis was run between the two subgroups. Based on a median (Me) value of vitamin C, E and β-carotene intake, a patient received ‘0’ (if the intake was <Me) or ‘1’ point (if the intake was ≥Me). Next the points were added and based on the sum result (min = 0, max = 3) the group was divided into group 1 (*n* = 43), with a lower vitamin intake (∑ = 0 or 1), and group 2 (*n* = 37), with a higher vitamin intake (∑ = 2 or 3). The two groups were identical with regard to sex profile (14% of males in each group).

### 2.3. Statistical Analysis

Data were verified for normality of distribution and equality of variances. Variables that did not meet the assumption of normality were analyzed with non-parametric statistics. Correlations between nutrient intake and age, BMI, WHR, lipid and antioxidant indices in plasma, and antioxidant parameters and CRP in saliva, were analyzed with the Spearman’s rank correlation coefficient. The Mann–Whitney test was used to compare the mean values of numerical variables between group 1 and group 2. The results of the quantitative variables were presented as a mean ± standard deviation (SD) and *p* < 0.05 was considered statistically significant for all analyses. The statistical analysis was performed using Statistica version 10 CSS software (StatSoft Polska Sp. z o.o., Kraków, Poland).

## 3. Results

### 3.1. Baseline Groups Characteristics

Detailed demographic, anthropometric and laboratory characteristics of studied groups are shown in Table 1. The two subgroups did not differ with regard to age. Over 1/3 of the group were diagnosed with obesity, and further 0.4 of the group with overweight. Visceral obesity was found in almost 3/4 of the group. Groups 1 and 2 were similar with regard to the anthropometric and lipid profile parameters except for TG: group 2 had a lower TG concentration (*p* < 0.01).

### 3.2. Antioxidant Indices and Salivary CRP

Table 2 presents the mean values of plasma and salivary antioxidant indices and salivary CRP. FRAS was decreased in group 2 (*r* = 2.9; *p* < 0.01) but no other intergroup differences were found for salivary or for plasma antioxidant parameters. Salivary CRP did not differ between groups.

### 3.3. Nutritional Characteristics

Generally, the study group was rather well nourished (71% covered the minimum demand for energy, 69% for total protein, 56% for dietary fiber, 41% for magnesium, 75% for zinc; according to the recommendations for the elderly). The percentage of the group with deficient vitamin E consumption of the AI standard was 54% (48% in females and 91% in males), while 23% were vitamin C deficient according to the EAR standard (23% in females and 18% in males). A detailed analysis of kind of fruit and vegetables common chosen by the study group indicated tomatoes, peppers, onion, potatoes, soup greens, cabbage, seasonal fruit (apples, raspberries, strawberries, cherries) as main sources of vitamin C and β-carotene (the average mass of fruit and vegetables jointly in about 2/3 of the study group was at range 600–900 g per day), with plant oils (mostly oilseed rape, olive oil) as sources of vitamin E.

Several similarities were found between the groups regarding the absolute values of the energy obtained from particular macronutrients intake (19% from proteins, 29% from fat and 51% from carbohydrates), for total fat, saturated and monounsaturated fatty acids, vitamin B_12_, sodium and manganese. Total energy (*p* < 0.001), total protein (*p* < 0.001), total carbohydrates (*p* < 0.01), sucrose (*p* < 0.001), dietary fiber (*p* < 0.001), polyunsaturated fatty acids (*p* < 0.01), cholesterol (*p* < 0.05) and water (*p* < 0.001) were significantly higher in group 2, as was the intake of some minerals (potassium, calcium, phosphorus, magnesium, iron, zinc, copper, iodine) and vitamins (B vitamins except for B_12_, vitamin A and D (*p* < 0.05)). As expected, vitamin C (84.5 ± 69.9 mg vs. 186.1 ± 66.9 mg), E (6.4 ± 2.2 mg vs. 10.7 ± 3.2 mg) and β-carotene intake (3627 ± 3773 µg vs. 6494 ± 3887 µg) were also significantly higher in group 2 (*p* < 0.001). After adjustment for nutritional density characteristics (calculation per 1000 kcal), significantly higher intake in group 2 remained for vitamin C, E, β-carotene, sucrose, dietary fiber, potassium, copper, vitamin B_6_ and folic acid.

### 3.4. Correlations for Antioxidant Indices and Salivary CRP in the Study Group (n = 80)

Age positively correlated only with salivary antioxidant indices: FRAS (*r* = 0.27), DPPHS (*r* = 0.23) and SUA (*r* = 0.28) but not with plasma antioxidants. Subjects with higher BMI had increased salivary CRP (*r* = 0.27), and those with higher TG had increased FRAP (*r* = 0.35) and UA (*r* = 0.23). Individuals with visceral obesity were characterized with higher UA (*r* = 0.30).

Lower calcium (*r* = −0.26), magnesium (*r* = −0.24) and vitamin B_12_ (*r* = −0.27) intake were related to higher salivary CRP, without their impact on any antioxidant parameters. Lower dietary fiber (*r* = −0.23), zinc (*r* = −0.27) and vitamin C (*r* = −0.26) intake corresponded only to higher FRAS. There were no other correlations between vitamins C, E or β-carotene intake and antioxidant indices or salivary CRP. Salivary CRP did not relate to any antioxidant indices, neither in saliva nor in plasma (*p* > 0.05). Instead, all plasma antioxidant indices (FRAP, DPPH, UA) correlated positively with their saliva analogues (FRAS, DPPHS, SUA) (*p* < 0.05).

## 4. Discussion

To the best of our knowledge, this is one of very few studies that assesses TAC by two different established methods in plasma (FRAP and DPPH) and in saliva (FRAS and DPPHS test) in a group of relatively healthy adults. It also performs the first simultaneous assessment of plasma and salivary UA and CRP in the context of dietary antioxidant intake. Our present findings indicate that a higher level of dietary vitamin C intake had an adverse effect on FRAS, but that the intake of other antioxidative vitamins from an habitual dietary intake did not affect the TAC or UA of plasma or saliva. Salivary CRP was not related to the identified level of antioxidant compounds in diet, but higher CRP levels were associated with lower calcium, magnesium and vitamin B_12_ consumption from the DFR. The nutritional status of group 2 was significantly superior to group 1 but generally the antioxidant status of both groups, besides FRAS index, was comparable. Also salivary CRP concentration, regardless of the combined vitamins C, E and β-carotene intake difference, was at a similar level in each group.

The knowledge about positive effect of dietary vitamins intake on good health conditions seems to be indisputable [32,33]. However their impact on the antioxidant potential and inflammatory indices is not so obvious. Recently, diet and CRP, in particular high sensitivity CRP (hs-CRP) are of increasing research interest. There are relatively few studies regarding salivary CRP, especially relating to habitual dietary intake, not to modified daily diet. Usually they concern a certain oral health or cardiac disorders or some dietary interventions. Salivary CRP as well as salivary TAC assessment in view of its noninvasive technique of samples collection seems to be appealing new research direction. In the study by Mazidi et al. [34] the increase in serum hs-CRP was associated with lower level of total dietary fiber and vitamins C, E, A intake (not as in the present study). The hs-CRP concentrations were likely modulated by dietary intake, including dietary sugar, polyunsaturated fatty acids, fiber and antioxidant intake. It is possible that higher PUFA intake may be related to the intensified oxidative stress and to the reduction of inflammation but the available data are full of discrepancies [35]. In our study, PUFA intake was significantly higher in group 2 with higher combined antioxidant vitamins intake but no significant correlation was found between plasma and salivary antioxidant indices nor salivary CRP and polyunsaturated fatty acids. Other reports also indicated that high intake of carotenoids and vitamin C, but not of vitamin E, seems to decrease the level of circulating hs-CRP [36]. In a crossover intervention by Valtueña et al. [37] plasma CRP decreased during the high-TAC diet. Instead, Stringa et al. [38] assessed whether total dietary antioxidant capacity (assessed by dietary FRAP) and serum UA were associated with low-grade chronic inflammation expressed as serum hs-CRP. The results, similarly as in our paper, demonstrated no association between dietary FRAP and hs-CRP levels but contrary to our findings increased levels of UA were observed in subjects with higher levels of hs-CRP. Zhang et al., identified that applying standard diet recommended by guidelines and high fruit and soybean products diet intervention yielded no different effects on serum UA [39].

Data regarding the influence of the dietary antioxidant compounds in DFR on antioxidant parameters, particularly those associated with saliva, is also scarce [40,41,42]. Stedile et al. [40] reported a positive correlation between dietary TAC, including vitamin C and polyphenols, and plasma TAC in healthy young women. Presumably, the endogenous defenses were fully functional in young subjects. Kamodyová et al. [6] reported that single intake of vitamin C (250 mg) had a positive influence on TAC in healthy participants. A study by Carrión-García et al. [43] in a group of healthy volunteers assessed the relationship between non-enzymatic antioxidant capacity (NEAC) estimated by two different dietary assessment methods (FRAP and trolox equivalent antioxidant capacity) and NEAC plasma levels: statistically significant but relatively weak, positive correlations were found between dietary FRAP (either derived from the food frequency questionnaire, or the 24-h recall) and plasma FRAP, particularly in the fruit and vegetables food groups. As the optimal TAC level for the human body is unknown, our results suggesting a lack of relationship between antioxidative dietary vitamin intake and most of the plasma and salivary antioxidant parameters cannot reduce the significance of habitual dietary intake solely on the basis of its failure to modulate antioxidant potential in vivo. Perhaps considering that it is desirable for human body to have a high TAC level, this area should be investigated further.

An unexpected negative correlation between dietary vitamin C intake and FRAS should also be explored. Sinha et al. [44] reported a positive correlation between dietary vitamin C intake and plasma ascorbic acid (AA) level, as well as some interrelationships between various plasma antioxidants: for instance, a positive association between β-carotene and α-tocopherol, and an inverse one between plasma AA and plasma UA. This observation was similar to another finding in which serum UA decreased in elderly subjects after they were supplemented with high doses of vitamin C [45]. Strawberries added to the usual diet as a source of vitamin C did not increase fasting non-urate plasma antioxidant activity [46]. Wang et al. [7] reported that plasma TAC (determined by VCEAC—vitamin C equivalent antioxidant capacity) was positively associated with dietary intakes of γ-tocopherol and β-carotene, as well as with plasma α-tocopherol and UA, in overweight and apparently healthy postmenopausal women. Our findings do not indicate any relationship between vitamin C consumption and the level of UA or SUA, but a negative relationship is indicated between vitamin C and FRAS (mainly contributed by SUA). At present we are not able to fully explain why this may be, i.e., a lower vitamin C intake is associated with only FRAS and not the other assessed salivary or plasma TAC indexes. Moreover, this correlation disappears in subgroups 1 and 2, but the negative trend remains.

As vitamin C may well contribute to eliminating UA, we may assume that higher vitamin C intake causes a decrease in FRAS, not in FRAP: in saliva, the FRAS test found SUA to be the predominant antioxidant (71.6%) while the FRAP method found the plasma UA to be less predominate (64.0%) [47]. Hence it is reasonable to assume that a link exists between vitamin C intake and salivary and plasma TAC level including SUA/UA that remains unknown for now.

On the other hand, our result might serve as an example of the theory of hormesis, according to which high antioxidant potential is an effect of an undesirable increase in prooxidant concentration, which is possible among the cases with lower vitamin C consumption. However, the question remains why this effect was visible only in saliva, only visible using the FRAS test, but did not appear in plasma. Several explanations are possible: one being the characteristics of methodology used (FRAS based on the ferrous ions reaction), and another the fact that the local prooxidant effect of vitamin C associated with the Haber-Weiss reaction may be stronger in the saliva environment than in plasma, resulting in intensified hydroxyl radical production and the loss of FRAS. Saliva is also more likely to be exposed to bacterial flora, probably generating reactive oxygen species. Wang et al., found that plasma TAC measured by VCEAC gave a better representation of plasma antioxidant levels than ORAC (oxygen radical absorbance capacity) or FRAP assay. However, TAC measured by FRAP correlated only with UA, while more correlations were found by VCEAC [7].

To avoid missing the possible resultant effect of various dietary antioxidative compounds, the different TAC assessment methods should be in future studies accompanied by the particular plasma antioxidant concentration assays.

It should be also noted that while both DPPH and FRAP tests measure the TAC, they reflect somewhat different physiological properties. As neither of the methods for TAC assessment measures all the antioxidants occurring in body fluids, the simultaneous use of both the FRAP and DPPH assays, in spite of their limitations, enhances the completeness and reliability of measurement. For instance Sinha et al., concluded that for people consuming large amounts of vitamin C, plasma AA is not an appropriate biomarker of dietary vitamin C [44].

Despite its strengths, such as its complexity (simultaneously applying two analytical methods in two body fluids, using a number of assessed parameters, the age-, sex- and anthropometric-comparable groups) the study also has some limitations, two being the limited number of subjects and the cross-sectional design of the study. It should be also noticed that our subjects were volunteers, who were probably healthier and fitter than a random sample, as well as more willing to participate in such studies. Nonetheless, bearing in mind the percentage of subjects deficient in vitamin E (54%) and vitamin C intake (23%) it may be assumed that, despite their mean vitamin C intake being more than adequate, the groups were not as well-nourished as could be expected. Moreover, the heterogeneity of the pharmacotherapy could interfere with the results. It was not feasible to find older subjects entirely free from common age-related ailments or using similar drugs and treatment regimens (the average senior suffers from 3–4 coexistent diseases). Nevertheless, the diseases diagnosed in our study group were in a stable phase and pharmacologically controlled.

## 5. Conclusions

A non-supplemented diet based on habitual dietary intake does not significantly affect plasma or salivary TAC and salivary CRP. The known health benefits of a natural, antioxidant-rich diet may be not related to plasma or salivary antioxidant potential. Further prospective studies are needed to examine these potential relationships.

## Figures and Tables

**Table 1:** Baseline characteristics of the study groups.

Variable	All (*n* = 80)	Group 1 (*n* = 43)	Group 2 (*n* = 37)
Age (years)	66.9 ± 4.3 (60.0 ÷ 79.0)	67.2 ± 4.3 (60.0 ÷ 77.0)	66.7 ± 4.4 (61.0 ÷ 79.0)
Body Mass Index (kg∙m^−2^)	29.3 ± 5.2 (21.4 ÷ 44.0)	29.8 ± 5.6 (21.4 ÷ 44.0)	28.7 ± 4.8 (22.6 ÷ 39.1)
Waist circumference (cm)	92.3 ± 12.9 (71.5 ÷ 130.0)	94.2 ± 13.7 (71.5 ÷ 130.0)	90.0 ± 11.5 (72.0 ÷ 123.0)
Waist-to-Hip Ratio	0.87 ± 0.09 (0.71 ÷ 1.07)	0.88 ± 0.09 (0.71 ÷ 1.07)	0.86 ± 0.09 (0.74 ÷ 1.07)
Total Cholesterol (mg∙dL^−1^)	182.2 ± 36.6 (100.5 ÷ 285.3)	177.6 ± 37.2 (100.5 ÷ 247.2)	187.6 ± 35.5 (119.7 ÷ 285.3)
LDL-Cholesterol (mg∙dL^−1^)	114.6 ± 33.3 (45.7 ÷ 196.7)	108.0 ± 33.9 (45.7 ÷ 172.5)	122.3 ± 31.3 (59.1 ÷ 196.7)
HDL-Cholesterol (mg∙dL^−1^)	45.1 ± 13.3 (17.4 ÷ 78.3)	43.0 ± 13.7 (19.7 ÷ 76.9)	47.5 ± 12.6 (17.4 ÷ 78.3)
Triglycerides (mg∙dL^−1^)	123.3 ± 54.7 (30.5 ÷ 249.3)	138.0 ± 48.8 (48.4 ÷ 244.6)	106.1 ± 56.9 ^†^ (30.5 ÷ 249.3)

Data are presented as mean ± SD (min ÷ max). ^†^—*p* < 0.01 as compared to group 1.

**Table 2:** Plasma and salivary antioxidant indices and salivary CRP concentrations.

Plasma	All (*n* = 80)	Group 1 (*n* = 43)	Group 2 (*n* = 37)	Saliva	All (*n* = 80)	Group 1 (*n* = 43)	Group 2 (*n* = 37)
FRAP (mmol FeCl_2_∙L^−1^)	1.21 ± 0.21 (0.81 ÷ 1.80)	1.25 ± 0.23 (0.81 ÷ 1.80)	1.17 ± 0.17 (0.85 ÷ 1.63)	FRAS (mmol FeCl_2_∙L^−1^)	5.99 ± 2.81 (2.11 ÷ 19.08)	6.75 ± 3.18 (3.01 ÷ 19.08)	5.11 ± 2.00 ^†^ (2.11 ÷ 11.49)
DPPH (% reduction)	23.4 ± 5.8 (8.6 ÷ 35.6)	24.3 ± 6.2 (8.6 ÷ 35.6)	22.5 ± 5.2 (15.0 ÷ 34.4)	DPPHS (% reduction)	27.4 ± 14.5 (3.5 ÷ 68.9)	27.7 ± 14.0 (9.8 ÷ 68.1)	27.2 ± 15.3 (3.5 ÷ 68.9)
UA (mg∙dL^−1^)	4.47 ± 1.16 (1.69 ÷ 7.38)	4.54 ± 1.02 (1.96 ÷ 6.34)	4.39 ± 1.32 (1.69 ÷ 7.38)	SUA (mg∙dL^−1^)	9.15 ± 4.16 (0.42 ÷ 22.33)	9.96 ± 4.13 (4.33 ÷ 22.33)	8.06 ± 4.01 (0.42 ÷ 16.19)
CRP (mg∙dL^−1^)	<0.3	<0.3	<0.3	Salivary CRP (ng mL^−1^)	2.23 ± 1.86 (0.35 ÷ 8.82)	2.22 ± 1.90 (0.35 ÷ 7.90)	2.24 ± 1.83 (0.47 ÷ 8.82)

Data are presented as mean ± SD (min ÷ max). FRAP—Ferric Reducing Ability of Plasma; DPPH—2.2-diphenyl-1-picryl-hydrazyl test of plasma; UA—Uric Acid; CRP—C-reactive protein; FRAS—Ferric Reducing Ability of Saliva; DPPHS—2.2-diphenyl-1-picryl-hydrazyl test of saliva; SUA—Salivary Uric Acid. ^†^—*p* < 0.01 as compared to group 1.
